# Development and validation of a nomogram for pneumonia risk in burn patients with inhalation injury: a multicenter retrospective cohort study

**DOI:** 10.1097/JS9.0000000000001190

**Published:** 2024-02-09

**Authors:** Shijie Li, Dawei Li, Yalong Li, Xinzhu Liu, Yaoyao Song, Xiaoye Xie, Peng Luo, Huageng Yuan, Chuan’an Shen

**Affiliations:** a Senior Department of Burns and Plastic Surgery, The Fourth Medical Center of PLA General Hospital; b Medical School of Chinese PLA, Beijing, People’s Republic of China

**Keywords:** burn, inhalation injury, nomogram, pneumonia

## Abstract

**Background:**

Burn patients with inhalation injury are at higher risk of developing pneumonia, and yet there is no reliable tool for the assessment of the risk for such patients at admission. This study aims to establish a predictive model for pneumonia risk for burn patients with inhalation injury based on clinical findings and laboratory tests.

**Method:**

This retrospective study enrolled 546 burn patients with inhalation injury. They were grouped into a training cohort and a validation cohort. The least absolute shrinkage and selection operator (LASSO) regression analysis and binary logistic regression analysis were utilized to identify risk factors for pneumonia. Based on the factors, a nomogram for predicting pneumonia in burn patients with inhalation injury was constructed. Areas under the receiver operating characteristic curves (AUC), calibration plots, and decision curve analysis (DCA) were used to evaluate the efficiency of the nomogram in both the training and validation cohorts.

**Results:**

The training cohort included 432 patients, and the validation cohort included 114 patients, with a total of 225 (41.2%) patients experiencing pneumonia. Inhalation injury, tracheal intubation/tracheostomy, low serum albumin, and high blood glucose were independent risk factors for pneumonia in burn patients with inhalation injury and they were further used to build the nomogram. The AUC of the nomogram in the training and validation cohorts were 0.938 (95% CI: 0.917–0.960) and 0.966 (95% CI: 0.931–1), respectively. The calibration curve for probability of pneumonia showed optimal agreement between the prediction by nomogram and the actual observation, and the DCA indicated that the constructed nomogram conferred high clinical net benefit.

**Conclusion:**

This nomogram can accurately predict the risk of developing pneumonia for burn patients with inhalation injury, and help professionals to identify high-risk patients at an early stage as well as to make informed clinical decisions.

## Introduction

Highlights

- The severity of inhalation injury, tracheal intubation/tracheostomy, low serum albumin, and high blood glucose were independent risk factors for pneumonia in burn patients with inhalation injury.
- This study is the first to apply a nomogram to predict the risk of pneumonia in burn patients with inhalation injury.
- The nomogram demonstrated satisfactory discriminative ability for identifying pneumonia and nonpneumonia patients in both cohorts.

Inhalation injury refers to respiratory impairment caused by hot air, smoke, chemicals, and other substances. It is one of the most common complications in burn patients and a major cause of mortality in this population, with an incidence of 10–20% in hospitalized burn patients^1,2^. The most common complication following inhalation injury is pneumonia, which involves a series of pathophysiological changes, including the activation of the host inflammatory response due to direct thermal injury, direct lung damage, damage to airway ciliated cells caused by chemical irritation, impairment of lung immune function, impaired surfactant production, compromised mucociliary clearance, and reduced lung macrophage function^1^. Researches have shown that burn patients with inhalation injury have an incidence of ~37% of pneumonia, while patients with inhalation injury requiring ventilatory support have a higher incidence of pneumonia, up to 50%^3,4^. Furthermore, burn patients with inhalation injury who developed pneumonia have an increased mortality rate, rising from 40 to 60%^5^. Though pneumonia is not an independent predictor of mortality, it significantly increases the duration of mechanical ventilation and is a high-risk complication in burn patients with inhalation injury^6^.

The occurrence of pneumonia in burn patients worsens the condition and exacerbates lung injury, adversely affecting treatment outcomes. Therefore, early identification of pneumonia is crucial to the management of burn patients. Clinically, the Clinical Pulmonary Infection Score (CPIS) has been widely used to enhance the specificity of pneumonia diagnosis. However, the performance of CPIS would be limited in burn patients due to the systemic inflammatory responses postburn^7,8^. In recent years, there is no progress on establishing pneumonia prediction models based on risk factors affecting the development of pneumonia in burn patients with inhalation injury.

This study utilized the least absolute shrinkage and selection operator (LASSO) regression and multiple logistic regression analysis to determine variables, and found that patients at higher risk of developing pneumonia had lower serum albumin, higher blood glucose levels within 48 h of admission, more severe inhalation injury, and underwent tracheal intubation/tracheostomy. Based on these indicators, a nomogram was established and validated to provide individualized prediction of pneumonia in burn patients with inhalation injury.

## Methods

### Patient selection and data collection

Data were retrospectively collected from January 2015 to December 2022 from 432 burn patients with inhalation injuries treated at A hospital: the Fourth Medical Center of Chinese PLA General Hospital (as the training cohort), and from January 2016 to December 2022 from 114 burn patients with inhalation injuries treated at B hospital: the 969 Hospital of the Joint Logistics Support Force of the Chinese PLA (as the validation cohort). Included patients were aged >18 years and all had concomitant inhalation injuries. Exclusion criteria were: 1) pre-existing pneumonia or severe cardiopulmonary diseases upon admission; 2) diabetic patients; 3) severe liver disease, hepatic insufficiency, and hypoalbuminemia; 4) patients with incomplete medical records. Data meeting the inclusion criteria were collected to establish a database for subsequent research. This research protocol was approved by the ethics committees of A and B hospital: the Fourth Medical Center of Chinese PLA General Hospital and the 969 Hospital of the Joint Logistics Support Force of the Chinese PLA. The work has been reported in line with the strengthening the reporting of cohort, cross-sectional, and case–control studies in Surgery (STROCSS) criteria^9^.

Demographic data for the enrolled patients include age, sex, BMI, smoking history; clinical data include total burn surface area (TBSA), burn depth, burn index (BI), degree of inhalation injury, whether tracheal intubation/tracheostomy was performed, and the occurrence of pneumonia during hospitalization; laboratory data include white blood cell count (WBC), serum albumin (ALB), blood glucose (Glu), and fibrinogen (FIB) within 48 h of admission. These were the key variables considered in this study.

### Diagnostic criteria

The primary outcome of the study was the occurrence of pneumonia during hospitalization in burn patients with inhalation injury. Pneumonia was confirmed by clinical diagnosis and etiological diagnosis. Clinical diagnostic criteria: (1) Chest radiography or CT shows new or progressive infiltrates, consolidation, or ground-glass opacities; with (2) Body temperature >39°C or <36.5°C; progressive tachycardia, adult heart rate >110 beats per minute; or (3) purulent respiratory secretions, or both. Etiological diagnostic criteria: (1) laboratory test of the lower respiratory tract secretions shows that: neutrophil count >25 cells/low-power field, epithelial cell count <10 cells/low-power field, or a ratio of >2.5:1); and (2) isolation of pathogens through bronchoalveolar lavage, or from lung tissue or sterile body fluid cultures, that are consistent with clinical manifestations^10^. The diagnosis of inhalation injury was primarily based on fiberoptic bronchoscopy or laryngoscopy results, all of which were conducted by experienced physicians. Inhalation injuries were graded into three degrees: mild: above the glottis, including injuries to the nose, pharynx, and glottis; moderate: above the tracheal carina, including injuries to the pharynx, larynx, and trachea; severe: below the bronchi, including injuries to the bronchi and lung parenchyma^11^.

### Statistical analysis

Data statistical analysis was performed using R language (4.3.0) and SPSS (26.0.0) software. Categorical data were presented as counts (%) and analyzed using the *χ* ^2^ test or Fisher’s exact test. Continuous variables were expressed as median (interquartile range), as appropriate. Parametric test (*t*-test) and nonparametric test (Mann–Whitney *U* test) were used for continuous variables with or without normal distribution, respectively.

Variables with missing values >15% were directly deleted, and the remaining missing values were imputed using multiple imputation. Subsequently, we employed LASSO regression method to screen for risk factors for pneumonia in burn patients with inhalation injuries. The selected variables were subjected to binary logistic regression analysis to establish a predictive model. The model’s discriminative ability was evaluated based on the receiver operating characteristic (ROC) curve, and the area under the ROC curve (AUC) was calculated. Model calibration was assessed using the calibration curve, visually presenting the results of the Hosmer–Lemeshow test. Decision curve analysis (DCA) was performed to evaluate the net clinical benefits. A value of *P*<0.05 was considered statistically significant.

## Results

### Demographics and clinical characteristics

**Figure 1:**
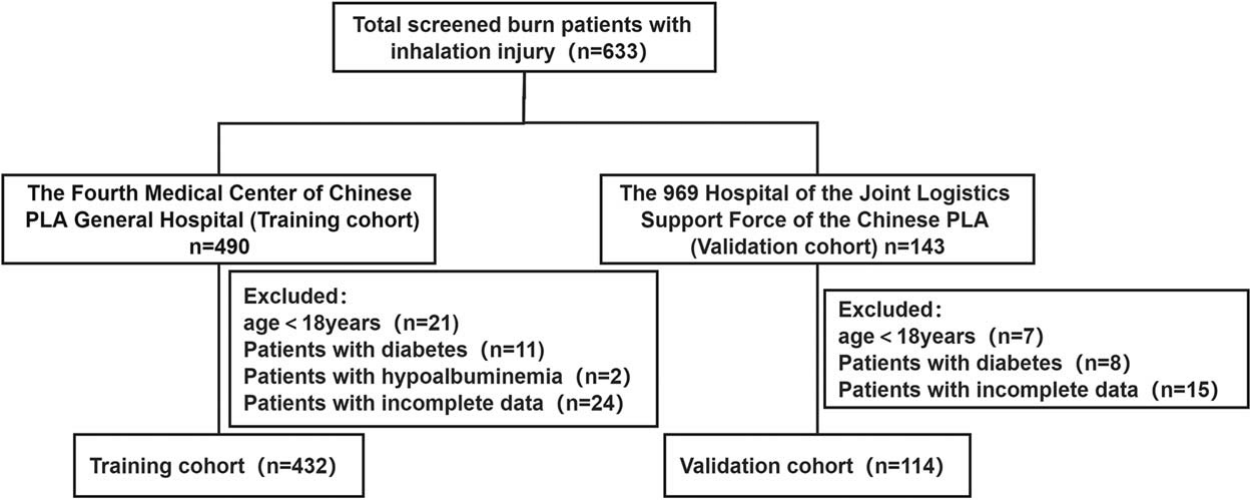
Flowchart of study participants in the training and validation cohorts.

The selection of the study population was shown in Figure 1. The training cohort included a total of 432 burn patients with inhalation injury, among whom 341 were male (78.9%) and 91 were female (21.1%). The median age was 42 years. The median TBSA was 40.00% (range, 16.00–70.00%), and the median BI was 25.50 (range, 8.13–55.00). All 432 patients were diagnosed with inhalation injuries upon admission, with 240 classified as mild (55.5%), 109 as moderate (25.2%), and 83 as severe (19.2%). There were 183 cases of pneumonia in training cohort, with an incidence of 42.3%, and there were 42 cases of pneumonia in validation cohort, with an incidence of 36.8%. The comparison of the clinical and demographic characteristics between the training and validation cohorts was shown in Table 1. Significant differences were observed in BMI, TBSA, and size of full thickness burn, total bilirubin, uric acid, lymphocyte count, hematocrit, neutrophil-to-lymphocyte ratio (NLR), platelet-to-lymphocyte ratio (PLR), systemic inflammation response index (SIRI), and fibrinogen between the two cohorts (*P*<0.05), while no significant differences of other variables were found (*P*>0.05).

**Table 1:** Comparison of variable characteristics between training cohort and validation cohort.

	Training cohort (*n*=432)			Validation cohort (*n*=114)			
	Pneumonia	No Pneumonia	*P* ^a^	Pneumonia	No pneumonia	*P* ^a^	*P* ^b^
Age (years)	40 (30–52)	43 (31–54)	0.102	42 (30–53)	46 (36–55)	0.312	0.07
Sex, No. (%)			0.729			1.000	0.093
Male	143 (78.1)	198 (79.5)		36 (86)	62 (86)		
Female	40 (21.9)	51 (20.5)		6 (14)	10 (14)		
BMI (Kg/m^2^)	24.30 (21.26–26.73)	24.69 (22.06–27.34)	0.235	24.22 (21.25–26.13)	23.42 (21.22–25.34)	0.340	0.018
Smoking history, No. (%)			0.012			1.000	0.978
Yes	70 (51.1)	67 (48.9)		13 (31)	23 (32)		
No	113 (38.3)	182 (61.7)		29 (69)	49 (68)		
TBSA (%)	70.00 (40.00–90.00)	25.00 (10.00–44.50)	＜0.001	70.00 (31.75– 90.00)	16.50 (10.00–30.25)	＜0.001	0.049
Burn depth							
Mixed thickness (%)	10.00 (4.00–20.00)	10.00 (5.00–22.00)	0.156	11.00 (5.00–26.50)	12.00 (6.00–20.00)	0.934	0.380
Full thickness (%)	41.00 (20.00–77.00)	5.00 (0.50–16.50)	＜0.001	40.00 (12.25–80.00)	3.00 (0.00–10.00)	＜0.001	0.021
BI	55.00 (30.00–80.00)	14.00 (5.00–30.00)	＜0.001	52.25 (24.38–85.38)	10.50 (5.50–20.00)	＜0.001	0.109
Inhalation injury grade, No. (%)			＜0.001			＜0.001	0.052
Mild	40 (21.9)	200 (80.3)		2 (5)	48 (67)		
Moderate	66 (36.1)	43 (17.3)		12 (29)	20 (28)		
Severe	77 (42.1)	6 (2.4)		28 (67)	4 (6)		
Tracheal intubation/tracheostomy, No. (%)			＜0.001			＜0.001	0.339
Yes	129 (88.4)	17 (11.6)		39 (93)	5 (7)		
No	54 (18.9)	232 (81.1)		3 (7)	67 (93)		
Total protein (g/l)	51.00 (43.90–57.70)	62.30 (55.20–67.30)	＜0.001	51.05 (47.60–55.75)	61.40 (57.58–65.73)	＜0.001	0.857
Albumin (g/l)	27.70 (24.00–31.50)	38.20 (33.10–41.85)	＜0.001	28.60 (24.90–29.58)	36.70 (34.27–38.50)	＜0.001	0.985
Globulin (g/l)	23.40 (19.40–27.10)	23.90 (21.05–26.95)	0.112	23.45 (21.40–26.10)	25.35 (22.75–27.27)	0.12	0.188
Blood glucose (mmol/l)	10.15 (7.66–12.91)	6.66 (5.75–7.86)	＜0.001	9.90 (8.62–11.90)	6.44 (5.60–7.60)	＜0.001	0.393
ALT (U/l)	33.90 (23.30–46.40)	25.00 (17.15–38.00)	＜0.001	36.00 (20.25–41.00)	25.00 (18.00–35.00)	0.052	0.552
TBil (μmol/l)	20.80 (14.40–33.10)	17.10 (12.80–24.10)	＜0.001	20.50 (12.45–24.97)	11.50 (9.25–19.25)	0.002	＜0.001
BUN (mmol/l)	6.32 (5.20–8.30)	5.10 (3.94–6.60)	＜0.001	6.20 (5.03–8.02)	5.30 (4.15–6.50)	0.005	0.793
Scr (μmol/l)	72.00 (58.00–92.30)	65.70 (55.66–77.45)	0.005	70.00 (57.00–83.75)	65.00 (57.50–77.00)	0.155	0.797
UA (μmol/l)	266.90 (190.00–357.50)	306.00 (228.70–377.10)	0.006	371.50 (272.50–457.50)	295.50 (203.00–394.00)	0.035	0.042
WBC (×10^9^/l)	18.89 (11.10–28.20)	13.97 (10.03–19.81)	＜0.001	25.30 (17.90– 30.03)	13.88 (10.24–17.73)	＜0.001	0.118
NEUT (×10^9^/l)	16.37 (9.54–25.05)	12.00 (7.65–17.40)	＜0.001	21.93 (14.53–25.77)	11.73 (8.02–15.40)	＜0.001	0.476
LYM (×10^9^/l)	1.06 (0.80–1.41)	1.27 (0.88–1.68)	0.014	1.98 (1.30–3.41)	1.31 (1.00–2.02)	0.004	＜0.001
MONO (×10^9^/l)	0.90 (0.60–1.48)	0.70 (0.45–1.01)	＜0.001	1.03 (0.66–1.65)	0.60 (0.41–0.89)	＜0.001	0.410
Ht (%)	47.00 (38.25–53.20)	45.70 (41.65–49.80)	0.524	51.70 (47.80–56.05)	46.85 (43.48–50.10)	＜0.001	0.001
Plt (×10^9^/L)	208.00 (127.50–277.00)	222.00 (179.50–271.00)	0.019	233.50 (201.25–300.50)	223.50 (172.00–261.75)	0.104	0.216
NLR	14.39 (8.93–23.24)	10.90 (5.54–16.99)	＜0.001	9.68 (5.27–17.39)	9.27 (4.23–15.09)	0.218	0.003
PLR	174.57 (103.99–257.71)	185.45 (129.42–272.86)	0.196	113.36 (79.04–172.86)	154.44 (104.61–221.29)	0.042	＜0.001
SII	2949.68 (1355–5693)	2257.34 (1107–3971)	0.018	2809 (1424–4681)	1635 (1016–3246)	0.055	0.089
SIRI	14.62 (5.77–30.98)	6.45 (2.82–13.51)	＜0.001	13.10 (4.92–23.27)	4.80 (1.70–12.62)	0.001	0.042
Fibrinogen (g/l)	3.51 (2.60–4.94)	2.97 (2.46–3.94)	0.001	2.10 (1.72–2.58)	2.16 (1.90–2.55)	0.385	＜0.001

a Comparisons were conducted between Pneumonia and No Pneumonia.

b Comparisons were conducted between the training cohort and validation cohort.

ALT, alaninetransaminase; BI, burn index; BUN, blood urea nitrogen; Ht, hematocrit; LYM, lymphocyte; MONO, monocyte; NEUT, Neutrophil count; NLR, neutrophil-to-lymphocyte ratio; PLR, platelet-to-lymphocyte ratio; Plt, platelet; Scr, serum creatinine; SII, systemic immune-inflammation index; SIRI, systemic inflammatory response index; TBil, total bilirubin; TBSA, total body surface area; UA, uric acid; WBC, white blood cells.

### Variable selection

**Figure 2:**
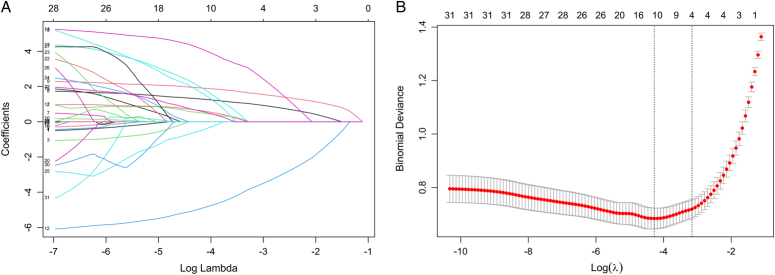
The variables filtering process of the Lasso regression. (A) LASSO coefficient profiles of the 31 baseline features; (B) Tuning parameter (λ) selection in the LASSO model used 20-fold cross-testing via minimum criteria.

In the training cohort, a total of 31 indicators were collected for each patient. First, variables were assigned values: group (pneumonia=1, nonpneumonia=0), smoking history (present=1, absent=0), degree of inhalation injury (mild=1, moderate=2, severe=3), tracheal intubation/tracheostomy (present=1, absent=0). These 31 indicators were then included in the LASSO regression analysis. Following the LASSO regression analysis (Fig. 2A, B), it was found that the severity of inhalation injury, tracheal intubation/tracheostomy, serum albumin, and blood glucose are significant predictive factors for pneumonia in burn patients with inhalation injury.

These four variables were incorporated into the binary logistic regression analysis, which revealed that the severity of inhalation injury (severe) (OR, 5.252; 95% CI: 1.607–17.162; *P*=0.006), tracheal intubation/tracheostomy (OR, 8.073; 95% CI: 3.578–18.212; *P*<0.001), serum albumin (OR, 0.867; 95% CI: 0.827–0.910; *P*<0.001), and blood glucose (OR, 1.221; 95% CI: 1.106–1.348; *P*<0.001) (Table 2) were significant independent predictors of pneumonia in burn patients with inhalation injury.

**Table 2:** Binary logistic regression analysis of pneumonia in burn patients with inhalation injury.

	Multivariate	
Variables	OR (95% CI)	*P*
Severity of inhalation injury		
Mild	Ref (–)	–
Moderate	1.817 (0.981–3.704)	0.100
Severe	5.252 (1.607–17.162)	0.006
Tracheal intubation/tracheostomy		
No	Ref (–)	–
Yes	8.073 (3.578–18.212)	＜0.001
Serum albumin	0.867 (0.827–0.910)	＜0.001
Blood glucose	1.221 (1.106–1.348)	＜0.001

### Construction and validation of the nomogram

**Figure 3:**
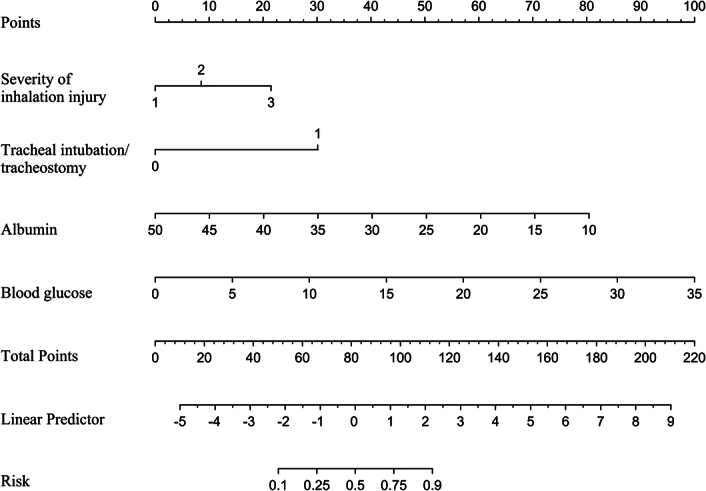
Nomogram of probability to develop pneumonia in burn patients with inhalation injury. To use the nomogram, draw an upward vertical line from each covariate to the points bar to calculate the number of points. Based on the sum of the covariate points, draw a downward vertical line from the total points line to calculate the probability of developing pneumonia.

Based on the results of the binary logistic regression analysis, the four variables were included in the nomogram (Fig. 3). Using the nomogram, each variable value can be assigned a corresponding score, and the total score for all variables can be vertically matched with the estimated probability of pneumonia in burn patients with inhalation injury. The higher the total score, the higher the risk of pneumonia. The optimal cut off for predicting pneumonia in the nomogram is 68.48 points (sensitivity: 91%, specificity: 84%).

**Figure 4:**
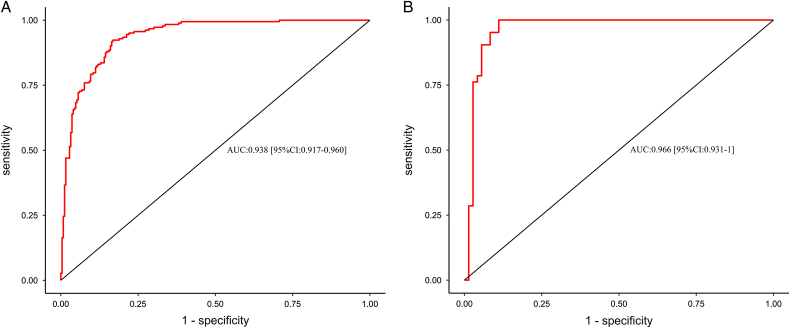
(A) ROC curve for the nomogram based on the training cohort. The AUC is 0.938; (B) ROC curve from the validation cohort and the AUC is 0.966. The estimate of AUC and its 95% CI are shown in the plots.

**Figure 5:**
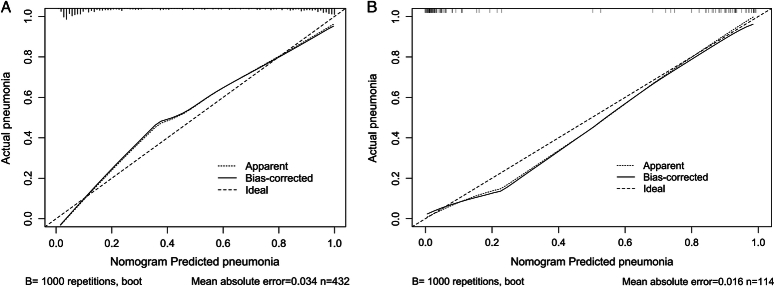
(A) Calibration curves of the nomogram for predicting pneumonia from the training cohort. The Hosmer–Lemeshow test had a *P*-value of 0.122 in the training cohort; (B) Calibration curves of the nomogram for predicting pneumonia from the validation cohort. The Hosmer–Lemeshow test had a *P*-value of 0.465 in the validation cohort.

**Figure 6:**
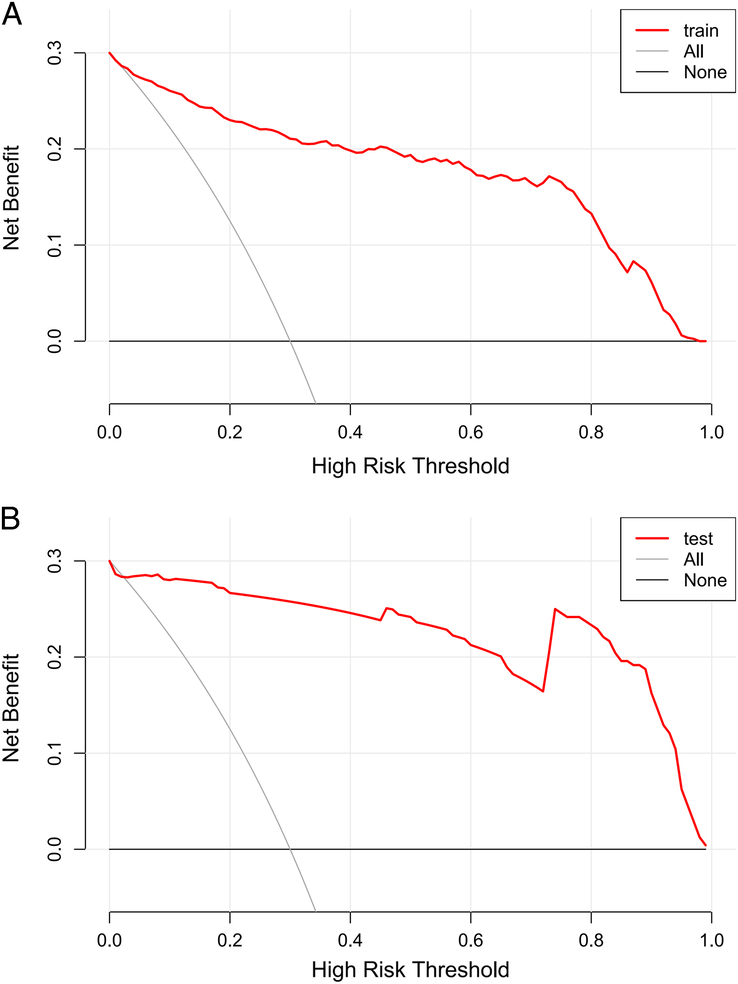
Decision curve analysis (DCA) of the nomogram. (A) The DCA curve of the training cohort; (B) The DCA curve of the validation cohort.

In both the training and validation cohorts, the nomogram exhibited excellent discriminative ability for differentiating between burn patients with inhalation injuries who developed pneumonia and those who did not, with AUC values of 0.938 (95% CI: 0.917–0.960) and 0.966 (95% CI: 0.931–1), respectively (Fig. 4A, B). The calibration curve demonstrated satisfactory consistency between the predicted probabilities of the nomogram and the actual probabilities, with Hosmer–Lemeshow test *P*-values of 0.122 and 0.465 in the training and validation cohorts, respectively (Fig. 5A, B). Furthermore, DCA curves were drawn for the training and validation cohorts to assess the clinical utility of the predictive model. DCA indicated that the nomogram conferred high clinical net benefit (Fig. 6A, B).

## Discussion

This study is the first to apply a nomogram to predict the risk of pneumonia in burn patients with inhalation injuries. We found that lower serum albumin levels and higher blood glucose within 48 h of admission, greater severity of inhalation injury, and the presence of tracheal intubation/tracheostomy were associated with a higher probability of pneumonia in burn patients with inhalation injuries. The nomogram established based on these findings demonstrated satisfactory discriminative ability for identifying pneumonia and nonpneumonia patients in both the training and validation cohorts, with AUC values of 0.938 and 0.966, respectively. DCA further indicated that the nomogram provided significant clinical net benefits. Additionally, the model’s external validation cohort also showed good performance. Moreover, the diagnosis of inhalation injury in both burn centers was made by experienced physicians to ensure diagnostic accuracy. The nomogram predictive model established in this study can be used in clinical practice to predict the risk of pneumonia in burn patients with inhalation injuries.

Inhalation injury has been repeatedly shown to be associated with an increased risk of pneumonia. Patients with inhalation injuries often exhibit restrictive ventilation, reduced pulmonary diffusion^12–14^, and disruption of lung immune function and mucociliary clearance capability due to injuries to the airway epithelium^1,15^, increasing their risk of developing pneumonia. Patients with severe inhalation injury exhibit a significantly increased production of pulmonary inflammatory mediators. Compared to burn patients without inhalation injury or with upper respiratory tract injury, the incidence of pneumonia and the blood culture positivity rate are higher in patients with lower respiratory tract injury^13,16^. This aligns with the conclusion in this study that a more severe degree of inhalation injury is associated with a higher risk of pneumonia in burn patients.

Additionally, severe inhalation injury patients often experience significant oropharyngeal swelling, necessitating the urgent establishment of an airway. The risk of pneumonia becomes evident during tracheal intubation or tracheostomy. Tracheal intubation can suppress cough reflex and mucociliary clearance, directly injure the tracheal mucosa during intubation, and create a pathway leading to the sterile lower respiratory tract^17^. Furthermore, as the disease progresses, patients who undergo tracheal intubation experience a breakdown of local barriers to infection and a weakened immune response. Oxygen-demanding Gram-negative rods and Staphylococcus aureus colonize the oral tracheal mucosa. Oral secretions contaminated with these bacteria accumulate in the oropharyngeal space above the tracheostomy tube cuff and below the vocal cords. If the cuff forms folds or cannot tightly adhere to the tracheal wall, bacterial-laden oral secretions can enter the lower respiratory tract through microaspiration, leading to pneumonia^18,19^. In this study, tracheal intubation/tracheostomy is an independent risk factor for pneumonia, which is consistent with the results of the systematic review and meta-analysis by Ding *et al*.^20^ Another single-center prospective and retrospective mixed-cohort study by Pawlik *et al*.^21^ also suggested a higher probability of pneumonia in patients undergoing percutaneous tracheostomy.

Albumin concentration and blood glucose are both laboratory indicators. In contrast to indicators like TBSA and the degree of inhalation injury that require clinical observation, they can further enhance the constructed nomogram’s prediction accuracy of pneumonia risk in the study. Hypoalbuminemia is a common complication in burn patients, especially in the severe ones. A study by Bandeira *et al*.^22^ suggested that burn patients with low serum albumin concentration (≤2.2 g/dl) are more prone to develop pulmonary infections. In addition, metabolic disturbances, especially hyperglycemia, are common complications in severely burned patients^23^. Ray *et al*.^24^ study showed that burn patients with hyperglycemia at admission had a higher incidence of bacteremia, pneumonia, and urinary tract infections. Our study excluded diabetic patients and focused on stress-induced hyperglycemia at admission. Burn injuries are accompanied by severe stress responses, which leads to the release of tumor necrosis factor-alpha, inhibiting tyrosine kinase and reducing tyrosine phosphorylation of insulin receptors^25^. In severe burns, IL-6 contributes to insulin resistance in the liver and skeletal muscles, acting upon insulin receptor substrates^26^. Liu *et al*.^27^ study showed that severe burns lead to pancreatic islet Sirt3 deficiency and increased mitochondrial ROS levels, which are associated with pancreatic islet cell apoptosis, resulting in pancreatic islet dysfunction. In this study, both blood glucose and albumin concentration within 48 h of admission were independent risk factors for pneumonia in burn patients with inhalation injury.

Whether the burn size is an independent risk factor for pneumonia in burn patients remains controversial. The increased risk of exposure to various pathogenic organisms in burn patients is attributed to the immune suppression caused by impaired innate and adaptive immune functions after severe burns^28^. In addition, the increase in extravascular lung water in critically ill patients is associated with the incidence of pulmonary diseases. Wang *et al*.^29^ study indicated a positive correlation between burn size and extravascular lung water index, and patients with a burn size >65.5% TBSA had a significantly increased incidence of pneumonia within 2 weeks after burn injury. However, in the studies by Tanizaki *et al*. and Liodaki *et al*., the incidence of pneumonia in burn patients with inhalation injury was not correlated with the increase of burn size^4,30^. This study also found that burn size is not an independent risk factor for pneumonia in inhalation injury patients.

There are some limitations to this study. First, the patient data for developing and validating the nomogram come from two different burn centers, and there were some statistical differences between the patient data in the validation and training cohorts. Second, the data collected did not include those of systemic toxicity resulted by cyanide or carbon monoxide inhalation, such as carboxyhemoglobin levels, or those reflecting the patients’ respiratory function, such as the PaO2/FiO2 ratio. Finally, as a retrospective study, further research involving more diverse populations is needed to better validate our nomogram model.

In summary, the nomogram we established can effectively identify and predict pneumonia in burn patients with inhalation injury. It can assist professionals in early identification of patients who may develop pneumonia and the formulation of appropriate treatment plans.

## Ethical approval

This study was approved by the ethics committee of the Fourth Medical Center of Chinese PLA General Hospital (approval number: 2021KY026-HS001) and the ethics committee of the 969 Hospital of the Joint Logistics Support Force of the Chinese PLA (approval number: NO.2023-06-12).

## Consent

This study is a retrospective cohort study. Informed consent was waived by our ethics committee because of the retrospective nature of our study, and which will not have adverse effects on the health and rights of patients.

## Sources of funding

This work is supported by the Program of National Natural Science Foundation of China (82072169, 82272279).

## Author contribution

S.L.: data curation, investigation, formal analysis, visualization, and writing – original draft; D.L.: data curation and visualization; Y.L.: data curation and investigation; X.L.: validation and investigation; Y.S.: resources and supervision; X.X.: resources; P.L.: data curation; H.Y.: writing – reviewing and editing; C.S. (corresponding author): conceptualization, methodology, supervision, and writing – reviewing and editing.

## Conflicts of interest disclosure

The authors declare that they have no financial conflict of interest with regard to the content of this report.

## Research registration unique identifying number (UIN)

1. Name of the registry: not applicable.
2. Unique identifying number or registration ID: not applicable.
3. Hyperlink to your specific registration (must be publicly accessible and will be checked): not applicable.

## Guarantor

Chuanan Shen and Shijie Li.

## Data availability statement

The datasets used and/or analyzed during the current study are available from the corresponding author on reasonable request.

## Provenance and peer review

Not commissioned, externally peer-reviewed.

## References

[1] Walker PF Buehner MF Wood LA. Diagnosis and management of inhalation injury: an updated review. Crit Care 2015;19:351. 26507130 10.1186/s13054-015-1077-4 PMC4624587

[2] Hogg G Goswamy J Khwaja S. Laryngeal trauma following an inhalation injury: a review and case report. J Voice 2017;31:388.e27–388.e31. 10.1016/j.jvoice.2016.09.017 27884557

[3] Edelman DA Khan N Kempf K. Pneumonia after inhalation injury. J Burn Care Res 2007;28:241–246. 17351439 10.1097/BCR.0B013E318031D049

[4] Tanizaki S Suzuki K. No influence of burn size on ventilator-associated pneumonia in burn patients with inhalation injury. Burns 2012;38:1109–1113. 22999207 10.1016/j.burns.2012.08.008

[5] Shirani KZ Pruitt BA Jr Mason AD Jr. The influence of inhalation injury and pneumonia on burn mortality. Ann Surg 1987;205:82–87. 3800465 10.1097/00000658-198701000-00015 PMC1492872

[6] Ronkar NC Galet C Richey K. Predictors and impact of pneumonia on adverse outcomes in inhalation injury patients. J Burn Care Res 2023;44:irad099–irad1297. 10.1093/jbcr/irad099 37352120

[7] Croce MA Swanson JM Magnotti LJ. The futility of the clinical pulmonary infection score in trauma patients. J Trauma 2006;60:523–527. 16531849 10.1097/01.ta.0000204033.78125.1b

[8] Pham TN Neff MJ Simmons JM. The clinical pulmonary infection score poorly predicts pneumonia in patients with burns. J Burn Care Res 2007;28:76–79. 17211204 10.1097/BCR.0b013E31802C88DB

[9] Mathew G Agha R for the STROCSS Group. STROCSS 2021: strengthening the reporting of cohort, cross-sectional and case-control studies in surgery. Int J Surg 2021;96:106165. 34774726 10.1016/j.ijsu.2021.106165

[10] Greenhalgh DG Saffle JR Holmes JH IV. American Burn Association Consensus Conference on Burn Sepsis and Infection Group. American Burn Association consensus conference to define sepsis and infection in burns. J Burn Care Res 2007;28:776–790. 17925660 10.1097/BCR.0b013e3181599bc9

[11] Burn and Trauma Branch of Chinese Geriatrics Society Guo F Zhu YS Huang J. [National experts consensus on clinical diagnosis and treatment of inhalation injury (2018 version)]. Zhonghua Shao Shang Za Zhi 2018;34:E004. 30440146 10.3760/cma.j.issn.1009-2587.2018.11.E004

[12] Charles WN Collins D Mandalia S. Impact of inhalation injury on outcomes in critically ill burns patients: 12-year experience at a regional burns centre. Burns 2022;48:1386–1395. 34924231 10.1016/j.burns.2021.11.018

[13] Monteiro D Silva I Egipto P. Inhalation injury in a burn unit: a retrospective review of prognostic factors. Ann Burns Fire Disasters 2017;30:121–125. 29021724 PMC5627549

[14] Won YH Cho YS Joo SY. Respiratory characteristics in patients with major burn injury and smoke inhalation. J Burn Care Res 2022;43:70–76. 34142710 10.1093/jbcr/irab085

[15] Jacob S Kraft R Zhu Y. Acute secretory cell toxicity and epithelial exfoliation after smoke inhalation injury in sheep: an electron and light microscopic study. Toxicol Mech Methods 2010;20:504–509. 20843269 10.3109/15376516.2010.511302

[16] Albright JM Davis CS Bird MD. The acute pulmonary inflammatory response to the graded severity of smoke inhalation injury. Crit Care Med 2012;40:1113–1121. 22067627 10.1097/CCM.0b013e3182374a67 PMC3290689

[17] Estes RJ Meduri GU. The pathogenesis of ventilator-associated pneumonia: I. Mechanisms of bacterial transcolonization and airway inoculation. Intensive Care Med 1995;21:365–383. 7650262 10.1007/BF01705418

[18] Keykha A Ramezani M Amini S. Oropharyngeal decontamination for prevention of VAP in patients admitted to intensive care units: a systematic review. J Caring Sci 2022;11:178–187. 36247039 10.34172/jcs.2021.029 PMC9526792

[19] Zhao T Wu X Zhang Q. Oral hygiene care for critically ill patients to prevent ventilator-associated pneumonia. Cochrane Database Syst Rev 2020;12:CD008367. 33368159 10.1002/14651858.CD008367.pub4 PMC8111488

[20] Ding C Zhang Y Yang Z. Incidence, temporal trend and factors associated with ventilator-associated pneumonia in mainland China: a systematic review and meta-analysis. BMC Infect Dis 2017;17:468. 28676087 10.1186/s12879-017-2566-7 PMC5496595

[21] Pawlik J Tomaszek L Mazurek H. Risk factors and protective factors against ventilator-associated pneumonia-a single-center mixed prospective and retrospective cohort study. J Pers Med 2022;12:597. 35455713 10.3390/jpm12040597 PMC9025776

[22] Bandeira NG Barroso MVVS Matos MAA. Serum albumin concentration on admission as a predictor of morbidity and mortality in patients with burn injuries. J Burn Care Res 2021;42:991–997. 33481997 10.1093/jbcr/irab004

[23] Rehou S Mason S Burnett M. Burned adults develop profound glucose intolerance. Crit Care Med 2016;44:1059–1066. 26934145 10.1097/CCM.0000000000001605 PMC4868799

[24] Ray JJ Meizoso JP Allen CJ. Admission hyperglycemia predicts infectious complications after burns. J Burn Care Res 2017;38:85–89. 27355659 10.1097/BCR.0000000000000381

[25] Badoiu SC Miricescu D Stanescu-Spinu II. Glucose metabolism in burns-what happens? Int J Mol Sci 2021;22:5159. 34068151 10.3390/ijms22105159 PMC8153015

[26] Jeschke MG Gauglitz GG Finnerty CC. Survivors versus nonsurvivors postburn: differences in inflammatory and hypermetabolic trajectories. Ann Surg 2014;259:814–823. 23579577 10.1097/SLA.0b013e31828dfbf1 PMC3732513

[27] Liu X Xie X Li D. Sirt3-dependent regulation of mitochondrial oxidative stress and apoptosis contributes to the dysfunction of pancreatic islets after severe burns. Free Radic Biol Med 2023;198:59–67. 36738799 10.1016/j.freeradbiomed.2023.01.027

[28] Moins-Teisserenc H Cordeiro DJ Audigier V. Severe altered immune status after burn injury is associated with bacterial infection and septic shock. Front Immunol 2021;12:586195. 33737924 10.3389/fimmu.2021.586195 PMC7960913

[29] Wang W Yu X Zuo F. Risk factors and the associated limit values for abnormal elevation of extravascular lung water in severely burned adults. Burns 2019;45:849–859. 30527647 10.1016/j.burns.2018.11.007

[30] Liodaki E Kalousis K Mauss KL. Epidemiology of pneumonia in a burn care unit: the influence of inhalation trauma on pneumonia and of pneumonia on burn mortality. Ann Burns Fire Disasters 2015;28:128–133. 27252611 PMC4837489

